# Effects of ingesting protein with various forms of carbohydrate following resistance-exercise on substrate availability and markers of anabolism, catabolism, and immunity

**DOI:** 10.1186/1550-2783-4-18

**Published:** 2007-11-12

**Authors:** Richard B Kreider, Conrad P Earnest, Jennifer Lundberg, Christopher Rasmussen, Michael Greenwood, Patricia Cowan, Anthony L Almada

**Affiliations:** 1 Exercise & Sport Nutrition Lab, Center for Exercise, Nutrition and Preventive Health, Baylor University, Waco, TX, USA; 2 Preventive Medicine Laboratory, Pennington Biomedical Research Center, Baton Rouge, Louisiana, USA; 3 St. Paul Heart Clinic, St. Paul, MN, USA; 4 College of Nursing, University of Tennessee Medical School, Memphis, TN, USA; 5 ImagiNutrition, Inc., Laguna Niguel, CA, USA

## Abstract

**Background:**

Ingestion of carbohydrate (CHO) and protein (PRO) following intense exercise has been reported to increase insulin levels, optimize glycogen resynthesis, enhance PRO synthesis, and lessen the immuno-suppressive effects of intense exercise. Since different forms of CHO have varying glycemic effects, the purpose of this study was to determine whether the type of CHO ingested with PRO following resistance-exercise affects blood glucose availability and insulin levels, markers of anabolism and catabolism, and/or general immune markers.

**Methods:**

40 resistance-trained subjects performed a standardized resistance training workout and then ingested in a double blind and randomized manner 40 g of whey PRO with 120 g of sucrose (S), honey powder (H), or maltodextrin (M). A non-supplemented control group (C) was also evaluated. Blood samples were collected prior to and following exercise as well as 30, 60, 90, and 120 min after ingestion of the supplements. Data were analyzed by repeated measures ANOVA or ANCOVA using baseline values as a covariate if necessary.

**Results:**

Glucose concentration 30 min following ingestion showed the H group (7.12 ± 0.2 mmol/L) to be greater than S (5.53 ± 0.6 mmol/L; p < 0.03); M (6.02 ± 0.8 mmol/L; p < 0.05), and C (5.44 ± 0.18 mmol/L; p < 0.0002) groups. No significant differences were observed among groups in glucose area under the curve (AUC) values, although the H group showed a trend versus control (p = 0.06). Insulin response for each treatment was significant by time (p < 0.0001), treatment (p < 0.0001) and AUC (p < 0.0001). 30-min peak post-feeding insulin for S (136.2 ± 15.6 *u*IU/mL), H (150.1 ± 25.39 *u*IU/mL), and M (154.8 ± 18.9 *u*IU/mL) were greater than C (8.7 ± 2.9 *u*IU/mL) as was AUC with no significant differences observed among types of CHO. No significant group × time effects were observed among groups in testosterone, cortisol, the ratio of testosterone to cortisol, muscle and liver enzymes, or general markers of immunity.

**Conclusion:**

CHO and PRO ingestion following exercise significantly influences glucose and insulin concentrations. Although some trends were observed suggesting that H maintained blood glucose levels to a better degree, no significant differences were observed among types of CHO ingested on insulin levels. These findings suggest that each of these forms of CHO can serve as effective sources of CHO to ingest with PRO in and attempt to promote post-exercise anabolic responses.

## Background

It has been recommended that athletes ingest CHO and PRO following exercise in order to enhance glycogen resynthesis, promote an anabolic hormonal environment, enhance PRO synthesis, and/or lessen the immuno-suppressive effects of intense exercise [1-5]. These recommendations are based on findings that ingestion of CHO following exercise increases insulin levels promoting glycogen restoration [6-10]. Additionally, increasing insulin levels following exercise optimizes an anabolic hormonal environment and can serve as a potent stimulator of PRO synthesis pathways [8,11-13]. For example, Zawadzki and associates [10] reported that adding PRO to a post-exercise CHO supplement promoted a greater increase in insulin levels and glycogen restoration. Chandler and colleagues [14] reported that ingesting CHO and CHO/PRO immediately following exercise promoted a greater increase in insulin concentrations than those consuming PRO only or control groups. Additionally, that subjects ingesting the CHO/PRO-supplement following exercise had a greater increase in growth hormone than control subjects and those ingesting PRO alone [14]. The authors suggest that this increase in insulin and growth hormone concentration may facilitate a more favorable environment for recovery than CHO alone [5,14]. Further, Kraemer and coworkers [15] found that ingestion of CHO and PRO two hours before, immediately following, and during three consecutive days of resistance-training increased blood glucose, insulin, growth hormone, and IGF-1 to a greater degree than a placebo. Others studies have reported that the provision of PRO or amino acids prior to and/or following exercise stimulates PRO synthesis [3,12,16-22]. Consequently, there is considerable evidence to support recommendations that athletes should ingest CHO and PRO following exercise in order to optimize glycogen resynthesis, promote an anabolic hormonal environment, and increase PRO synthesis [1-5].

While a number of studies have evaluated the effects of providing different forms of PRO and amino acids on recovery and/or training adaptations [12,16,23-27], few studies have evaluated whether the provision of different forms of CHO with PRO influences recovery indices. The type of CHO ingested with PRO is an important consideration because the glycemic index of a CHO may enhance glycogen storage and/or anabolic responses to exercise by promoting a greater glucose and insulin response [28,29]. Theoretically, ingesting high glycemic index (GI) CHO would promote the greatest increase in insulin levels, glycogen resynthesis, and PRO synthesis. On the other hand, since ingestion of CHO with PRO has been reported to promote greater increases in insulin [9,28,30,31], it is possible that the insulin response may be maximized regardless of GI of the CHO ingested. Moreover, ingesting different forms of CHO may have other physiological influences that may optimize recovery. For example, although ingesting a high GI CHO may promote a rapid increase in insulin levels, it may be more advantageous to ingest a moderate GI CHO that may allow for a more gradual increase in glucose and insulin over time much like findings indicating that different types of PRO and/or amino acids have varying effects on anabolism and catabolism [32] as well as training adaptations [24,25,27]. The purpose of this study was to examine whether the type of CHO ingested with PRO following resistance-exercise has any effects on blood glucose availability, insulin levels, markers of anabolism and catabolism, and/or general immune markers during recovery.

## Methods

### Subjects

Forty subjects (19 males and 21 females) volunteered to participate in this study. All subjects had participated in at least one year of resistance training prior to testing and were informed of the possible risks of the investigation before giving their written and informed consent previously approved by the University of Memphis Institutional Review Board for the use of human subjects.

### Baseline testing

Prior to the start of this trial, a familiarization session was conducted to obtain one-repetition maximum (1RM) for each of nine Nautilus (*Nautilus, Inc., Vancouver, WA, USA*) weight-training exercises used during the experimental treatment. Exercises included the chest press, seated row, shoulder press, lat pull, leg extension, leg curl, biceps curl, triceps extension, and leg press. For the exercises in which 1RM was exceeded by the weights available on an individual machine, the Epley formula was used to predict 1RM based on the number of repetitions lifted at a given weight [33]. Rest periods between each 1 RM lifting attempt were not limited so that the subjects had adequate opportunity to perform to the best of their ability.

### Experimental protocol

Subjects reported to the testing laboratory after abstaining from exercise for 48-hours prior to testing and fasting overnight (~12 h). The time of day was standardized within a 2-h starting time for all subjects in order to minimize potential diurnal variation in hormonal concentrations. Upon arrival weight (kg) and height (cm) were recorded. Subjects then donated a pre-exercise baseline blood samples prior to starting the resistance-training workout. Each subject performed 3 sets of 10 repetitions at approximately 70% of 1 RM on nine exercises as described above. Each set of exercise was interspersed with a 2-minute rest period and research assistants monitored all sessions. If a participant could not complete the full 10 repetitions the weight was reduced so 10 repetitions could be completed during the following set of exercise. During each set, the weight lifted and the number of repetitions performed was recorded for each subject in order to calculate total lifting volume. Following the completion of the workout, subjects returned to the laboratory and donated a post exercise blood sample.

### Supplementation

After the post-exercise blood sample was obtained, subjects received in a double blind and randomized manner a CHO and PRO supplement containing 40 g of whey PRO with 120 g of sucrose (S), powdered honey (H), or maltodextrin (M). The remaining group served as a non-supplemented control group. These forms of CHO were selected because prior research in our lab demonstrated that ingesting 50 g of gel forms of these CHO's resulted in significantly different glucose and insulin profiles [34]. The H powder (*ADM Arkady, Olathe, KS, USA*) contained a 95% mixed CHO source containing fructose (31.5%), glucose (26%), wheat starch (25.3%), soluble fiber (12.5%) and maltose (4.7%). The supplements were similarly colored, flavored and packaged for double-blind administration by an independent food-packaging lab (*Paragon Labs, Torrance, CA, USA*). Research assistant's blended pre-measured volumes of the powder into 16 ounces of water to form a milk shake type drink. Subjects were given as much time as needed to ingest the supplements which typically was less than 5-min. Once the subject consumed the supplement, a timer was started and blood samples were taken at 30, 60, 90, and 120-min during recovery. A similar time frame for collection of blood samples was employed for the control group. Subjects remained seated during the recovery period. As blood samples were collected, subjects were asked to respond to a questionnaire assessing the severity of hypoglycemia, dizziness, fatigue, headache, and stomach upset they experienced throughout the experiment. Questions were asked on a scale of 0–10 with 0 having no symptoms and 10 being most severe.

### Blood analysis

During the pre-exercise baseline blood collection period, blood samples were obtained by a qualified nurse/phlebotomist using standardized venipuncture techniques. Following exercise, subjects were fitted with a 20G × 1" Jelco™ intravenous catheter (*Johnson & Johnson Medical, Arlington, TX, USA*). Once the catheter was inserted and stabilized, a locking luer male adapter plug with an intermittent injection site was then connected to the female end of the catheter. A Vacutainer™ needle (*Becton Dickinson and company, Franklin Lakes, NJ, USA*) was then attached to a Vacutainer holder in which the needle was inserted into the injection site of the adapter plug. Approximately 15 mL of venous blood was collected into two separate 6 mL labeled Vacutainer brand SST™ tubes and 5 mL into an EDTA Vacutainer tube (*Becton Dickinson and *Co.*, Franklin Lakes, NJ, USA*). Once the blood samples were obtained, approximately 2–3 mL of Bacteriostatic 0.9% sodium chloride (*Abbott Laboratories, North Chicago, IL, USA*) was infused into the catheter line using a syringe. Once the line was cleared with saline, the catheter was locked to prevent clotting of blood in the line between sampling intervals.

Blood samples were then centrifuged for 10 minutes at 3000 rpm in an Adams Physicians Compact Centrifuge (*Clay Adams, Parsippany, NJ, USA*). One of the SST tubes was divided into four labeled 2.0 mL Costar micro centrifuge tubes (*Corning Inc., Corning, NY, USA*) and frozen in a So-Low Ultra freezer (*Environmental Equipment, Cincinnati, OH, USA*) at -80°C for subsequent hormonal analysis. The second SST tube and an EDTA tube were sent to Quest Diagnostics (*Minneapolis, MN, USA*) for analysis of glucose, muscle and liver enzymes. Serum samples were assayed using a Technicon DAX model 96-0147 automated chemistry analyzer (*Technicon Inc. Terry Town, NY, USA*) following standard clinical procedures. Whole blood cell counts with percent differentials were run on whole blood samples using a Coulter STKS automated analyzer using standard procedures (*Coulter Inc., Hialeah, FL, USA*). These analyzers were calibrated daily to controls according to manufacturer's recommendations and federal guidelines for clinical diagnostic laboratories. Test to test reliability of performing these assays ranged from 2 to 6% for individual assays with an average variation of ± 3%. Samples were run in duplicate to verify results if the observed values were outside control values and/or clinical norms according to standard procedures. Blood samples were assayed for each variable at all data points with the exception that creatine kinase was only measured at baseline, following exercise, and 120 minutes following supplementation.

Frozen serum samples were assayed at the Exercise Biochemistry Lab at the University of Memphis using standardized spectrophotometric and enzymatic immunoassay procedures. Each sample was thawed only once and decoded only after all the analyses were completed. The quantitative measurement of insulin was determined using the DSL-10-1600 ACTIVE™ insulin Enzyme-Linked Immunosorbent (ELISA) Kit (*Diagnostic Systems Laboratories, Webster, TX, USA*). All insulin assays were completed in duplicate and read at 450 and 600 nm with an MRX Microplate Reader (*Dynatech Laboratories, Chantilly, VA, USA*) in ambient conditions averaging 27°C and 23% relative humidity. Outlying concentrations with coefficients of variability > 10% were removed to provide the best fitting curve. The intra-assay coefficient of variability for insulin measurements was 3.4% while the inter-assay coefficient of variability was 5.2%. The best fitting curve provided an r^2 ^value of ≥ 0.9916 for insulin. Testosterone and cortisol were assayed in the same manner as insulin using ACTIVE™ DSL-10-4000 and DSL-10-2000 Enzyme Immunoassay (EIA) kits, respectively. The testosterone and cortisol mean coefficient of variability was 2.6% and 2.3% respectively. Inter-assay coefficient of variability for testosterone and cortisol were 13.8% and 11%, respectively. The best fitting curve provided an r^2 ^value of ≥ 0.9996 for testosterone and ≥ 0.9668 for cortisol.

### Data analysis

Data were analyzed using Statview for Windows (SAS Institute, *Cary, NC*). A three-way multivariate analysis of variance (MANOVA) was utilized to examine gender × group × time interactions. As noted below, although some gender effects were observed, no significant group × time × gender differences were observed. Therefore, two-way repeated measures analysis of variance (ANOVA) was utilized to evaluate treatment, time, and treatment × time interactions. Least square significant difference (LSD) post-hoc analyses were used when significant ANOVA effects were observed. For values that where significant between groups at baseline, repeated measures ANCOVA analyses were employed. Consequent to randomization procedures, total lifting volume, fasting glucose and insulin concentrations were different between groups. Thus, covariate adjustments were made to the repeated measures analysis and AUC measures using these three variables. Delta concentrations were also calculated on post-exercise responses by subtracting 30, 60, 90, and 120-minute concentrations from the immediate un-fed/post-exercise concentration for glucose, insulin, testosterone and cortisol so as to determine integrated area under the curve (AUC) via trapezoidal rules. Data are presented as means and ± SEM.

## Results

### Subject demographics

Demographic data for each treatment group are presented in Table 1. Body mass adjusted 1 RM for the nine exercises were: chest press (0.83 ± .04 kg·kg^-1^), seated row (1.37 ± 0.06 kg·kg^-1^), shoulder press (0.70 ± 0.05 kg·kg^-1^), lat pull (0.79 ± 0.03 kg·kg^-1^), leg extension (1.0 ± 0.06 kg·kg^-1^), leg curl (0.51 ± 0.03 kg·kg^-1^), biceps curl (0.62 ± 0.03 kg·kg^-1^), triceps extension (0.66 ± 0.04 kg·kg^-1^) and leg press (2.0 ± 0.1 kg·kg^-1^). After adjustments were made during the exercise treatment in order to complete 3 sets of 10 repetitions per exercise, the mean percentage of maximum weight lifted during the weight training stimulus were as follows: chest press (66.6% ± 1.5), seated row (69.1% ± 1.1), shoulder press (62.1% ± 1.3), lat pull down (69.5% ± 0.7), leg extension (64.5% ± 2.0), leg curl (60.8% ± 2.3), biceps curl (64.3% ± 1.1), triceps extension (67.7% ± 1.0), and leg press (67.9% ± 1.3). The total lifting volume for each treatment group was: control (11,783 ± 1,618 kg), sucrose (11,447 ± 1,368 kg), honey (13,049 ± 1,320 kg), and maltodextrin (14,410 ± 2,006 kg). No significant differences were observed among groups in total lifting volume (p = 0.18).

**Table 1:** Demographic data

		**C**	**S**	**H**	**M**
**Age (y)**					
	x	20.9	24.0	23.3	24.7
	±	0.7	1.0	1.1	1.6
**Weight (kg)**					
	x	72.4	71.2	70.7	84.5
	±	5.9	4.0	4.8	7.1
**Height (cm)**					
	x	171.2	171.3	171.2	175.8
	±	3.4	5.0	3.7	3.6
**Training Volume (days/wk)**					
	x	4.2	4.2	4.1	3.7
	±	0.5	0.3	0.4	0.3
**Last meal (hrs)**					
	x	10.9	11.0	12.0	11.8
	±	0.8	0.7	0.5	0.5

x Represents mean value

± Represents standard error of mean

### Gender analysis

Gender analysis revealed that testosterone (p = 0.001) and the ratio of testosterone to cortisol (p = 0.001) values were significantly lower in women than men. However, no gender differences were observed in cortisol (p = 0.33). Likewise, no group × gender interactions were observed for cortisol (p = 0.57), testosterone (p = 0.77), or the ratio of testosterone to cortisol (p = 0.43). Moreover, no significant group × time × gender interactions were observed in cortisol (p = 0.10), testosterone (p = 0.88) or the ratio of cortisol to testosterone (p = 0.54). Therefore, data were analyzed by a two-way ANOVA and are presented as means for men and women combined.

### Glucose and insulin

**Figure 1:**
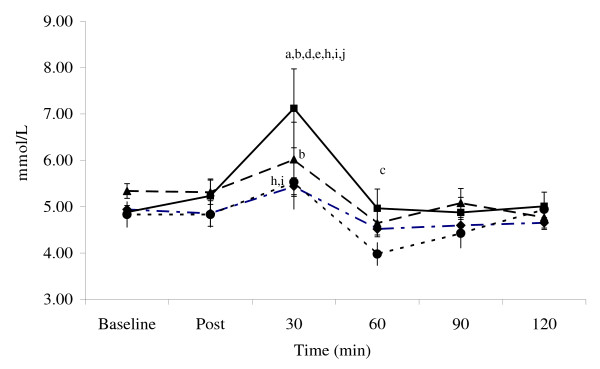
**Glucose responses**. Data (mean ± SE) represent individual treatment time measures for glucose (top panel) and insulin (bottom panel) for the control (diamond), sucrose (circle), honey (square), and maltodextrin (triangle) groups. Lower script values represent statistical significance where: a > post-exercise condition; b > control, c > honey, d > maltodextrin, e > sucrose, f < baseline, g < post-exercise, h > baseline. Other areas of significance are detailed in the "Results" section.

**Figure 2:**
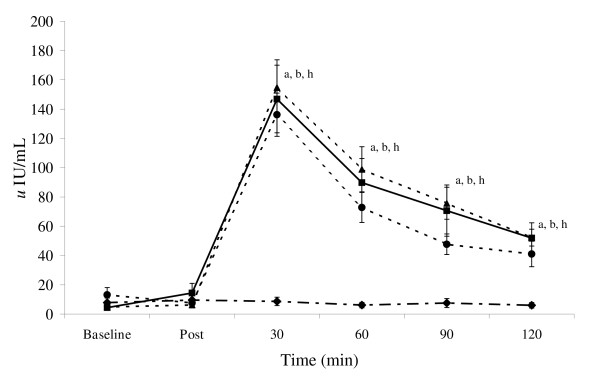
**Insulin responses**. Data (mean ± SE) represent individual treatment time measures for glucose (top panel) and insulin (bottom panel) for the control (diamond), sucrose (circle), honey (square), and maltodextrin (triangle) groups. Lower script values represent statistical significance where: a > post-exercise condition; b > control, c > honey, d > maltodextrin, e > sucrose, f < baseline, g < post-exercise, h > baseline. Other areas of significance are detailed in the "Results" section.

Figure 1 presents glucose while Figure 2 shows insulin concentrations observed during the experiment. Repeated measures ANCOVA revealed within (p < 0.01) and between group treatment effects for blood glucose concentration (p = 0.056). Within group post-hoc comparisons showed the H group glucose concentrations reached a significant peak 30-min following ingestion compared to baseline and post-workout concentrations (p < 0.05). Plasma glucose then declined to a lower concentration (i.e., versus 30-min) at 60, 90, and 120-min (p < 0.05). Glucose concentrations in the S group followed a similar post-feeding response, as 30-minute post ingestion concentrations were higher than 60 or 90-minute concentrations (p < 0.05), but not different than the immediate post workout/un-fed concentration.

**Figure 3:**
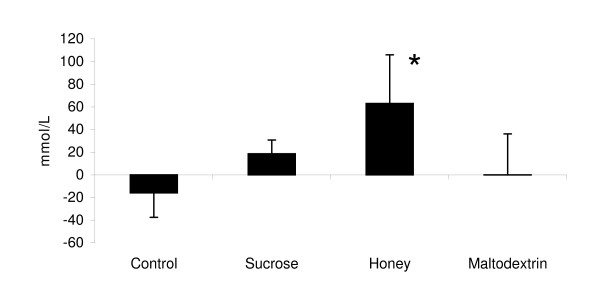
**Glucose AUC responses**. Data (mean ± SE) represent the change in AUC values observed following supplementation for glucose (top panel) and insulin (bottom panel). * represents (p < 0.05) difference from control.

**Figure 4:**
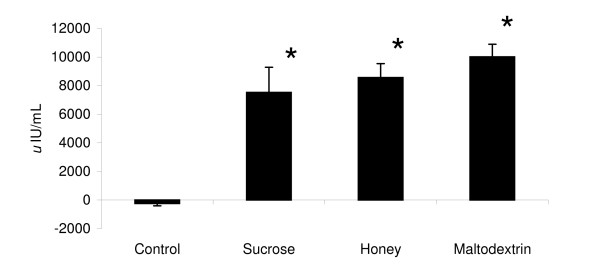
**Insulin AUC responses**. Data (mean ± SE) represent the change in AUC values observed following supplementation for glucose (top panel) and insulin (bottom panel). * represents (p < 0.05) difference from control.

Between-group treatment effects were also noted for plasma glucose concentrations (p < 0.056) as post-hoc analysis revealed 30 min glucose concentrations for the H group (7.12 ± 0.2 mmol/L) to be greater than S (5.53 ± 0.6 mmol/L; p < 0.03); M (6.02 ± 0.8 mmol/L; p < 0.05), and C (5.44 ± 0.18 mmol/L; p < 0.0002). At 60 min following ingestion, the H group was still greater than the S group (p < 0.02). The M group was also greater than C 30 min following ingestion (p < 0.03). No between group differences were observed at 90 and 120 min. Figure 3 shows AUC data for glucose while Figure 4 presents insulin AUC data. Blood glucose AUC values showed a significant treatment effect (p < 0.03) as post-hoc analysis showed the H treatment glucose concentration tending to be greater than C group values (p < 0.06).

Significant effects for time (p < 0.0001), treatment (p < 0.00001) and AUC (p < 0.0001) were observed in insulin values. Insulin concentrations (p < 0.05) and insulin AUC values (p < 0.05) were significantly higher than C values at 30, 60 90 120 min (p < 0.05) in the S, H, and M groups. 30-min peak post-feeding insulin for S (136.2 ± 15.6 *u*IU/mL), H (150.1 ± 25.3 *u*IU/mL), and M (154.8 ± 18.9 *u*IU/mL) were greater than C (8.7 ± 2.9 *u*IU/mL) as was the change in AUC after ingestion of the supplements (C -243 ± 162 *u*IU/mL; S 7,527 ± 883 *u*IU/mL; H 8,656 ± 1,758 *u*IU/mL; M 10,016 ± 974 *u*IU/mL). However, the type of CHO ingested with PRO did not significantly affect insulin response.

### Anabolic & catabolic hormones

Table 2 presents testosterone, cortisol and the testosterone to cortisol ratio data observed during the experiment. Significant time affects (p < 0.02) were observed for testosterone and cortisol concentrations. However, no significant group × time interactions were observed in testosterone, cortisol, or the ratio of testosterone to cortisol. Likewise, no significant differences were observed among groups in testosterone, cortisol, or the ratio of testosterone to cortisol AUC values.

**Table 2:** Anabolic and Catabolic Hormones

**Variable**	**Group**		**Pre**	**Post**	**30**	**60**	**90**	**120**	
**Testosterone **(nmol/L)	C	x	5.31	5.14	3.65	3.64	3.55	2.60	G p = 0.57
		±	1.37	1.38	.88	1.03	1.00	.93	T p = 0.02
	S	x	3.69	6.11	3.11	3.51	3.53	3.31	G × T p = 0.55
		±	1.78	3.29	1.38	2.13	2.08	1.77	
	H	x	3.02	2.73	2.41	2.33	2.35	2.50	
		±	0.54	0.44	0.36	0.32	0.31	0.25	
	M	x	2.27	2.62	2.09	2.04	1.82	1.51	
		±	0.67	0.85	0.71	0.60	0.51	0.40	
									
**Cortisol**	C	x	517	556	687	441	577	482	G p = 0.23
(nmol/L)		±	89	115	96	85	44	136	T p = 0.00
	S	x	448	397	526	569	489	294	G × T p = 0.71
		±	117	95	97	160	186	36	
	H	x	542	416	477	349	333	257	
		±	89	56	88	58	59	39	
	M	x	508	395	399	406	311	265	
		±	96	77	59	42	44	36	
									
**T/C Ratio**	C	x	2.4	2.4	1.9	2.6	2.3	3.3	G p = 0.10
		±	0.8	0.9	0.7	1.3	0.9	1.2	T p = 0.09
	S	x	1.8	2.7	2.0	1.7	2.0	1.9	G × T p = 0.19
		±	0.5	1.0	0.7	0.7	0.6	0.5	
	H	x	2.3	2.8	1.9	2.6	2.3	3.3	
		±	0.8	1.2	0.5	0.9	0.8	1.1	
	M	x	4.3	5.8	3.3	4.5	6.7	5.7	
		±	1.1	2.4	0.8	1.8	2.4	1.4	

x Represents mean value

± Represents standard error of mean

### Hepatorenal analysis

Table 3 presents creatinine, blood urea nitrogen (BUN), and the ratio of BUN to creatine results. Significant time effects were observed for each of these variables. Nutritional supplementation had no group × time effects on creatinine and BUN. However, a significant interaction was observed in the ratio of BUN to creatinine. Post-hoc analysis revealed that the BUN to creatinine ratio was significantly higher toward the end of recovery in the groups receiving CHO and PRO. However, no differences were observed among types of supplements investigated.

**Table 3:** Hepatorenal Variables

**Variable**	**Group**		**Pre**	**Post**	**30**	**60**	**90**	**120**	
**Creatinine **(mg/dL)	C	x	1.0	1.1	1.1	1.0	1.0	1.0	G p = 0.72
		±	0.1	0.1	0.1	0.1	0.1	0.1	T p = 0.00
	S	x	1.1	1.1	1.1	1.1	1.1	1.0	G × T p = 0.82
		±	0	0.1	0.1	0.1	0.1	0.1	
	H	x	1.1	1.2	1.1	1.0	1.0	1.0	
		±	0.1	0.1	0.1	0.1	0.1	0.1	
	M	x	1.2	1.3	1.2	1.1	1.0	1.1	
		±	0.1	0.1	0.1	0.1	0.1	0.1	
									
**BUN **(mg/dL)	C	x	13.3	13.7	13	12.9	12.9	12.9	G p = 0.90
		±	0.9	0.9	1.1	1.0	1.1	1.0	T p = 0.00
	S	x	14.3	13.9	13.9	14.6	16	15.8	G × T p = 0.98
		±	1.1	0.9	0.9	1	1.1	0.9	
	H	x	14.2	14	13.9	14.1	15.4	15.9	
		±	1.2	1.2	1.3	1.1	1.3	1.4	
	M	x	14.8	15	14.9	14.1	15.2	16.3	
		±	1.3	1.2	1.5	0.9	1.0	1.2	
									
**BUN/Creatinine Ratio**	C	x	12.8	13.1	12.3	12.6	12.5	12.6	G p = 0.70
		±	0.5	1.1	0.7	0.5	0.5	0.5	T p = 0.00
	S	x	13.8	13.4	13.3	14	14.7	16.0*	G × T p = 0.04
		±	1.3	1.9	1.1	1.1	0.9	1.2	
	H	x	13.6	11.8	12.6	14	15.1*	15.2*	
		±	1.0	0.9	1.0	1.1	1.2	1.2	
	M	x	12.7	11.6	12.6	13.3	15.1*	14.7	
		±	0.8	0.7	0.9	0.9	1.2	0.9	

x Represents mean value

± Represents standard error of mean

* p < 0.05 versus control

### Muscle and liver enzymes

Table 4 shows muscle and liver enzyme levels observed during the study. Significant time effects were observed for creatine kinase (CK) as the postexercise/un-fed and 120-minute post feeding concentrations were higher than baseline. Mean CK data increased from 136.8 ± 52.8 U/L at baseline to 264.3 ± 64.3 U/L after exercise and 264.0 ± 63.5 U/L after 120 min following nutritional supplementation (p < 0.001). No affects were noted for lactate dehydrogenase (LDH). Post exercise/un-fed aspartate aminotransaminase (AST) and alanine aminotransaminase (ALT) concentrations were significantly greater than pre-exercise values. However, AST and ALT levels declined thereafter. No significant group × time effects were observed in AST or ALT values among groups.

**Table 4:** Muscle and Liver Enzymes

**Variable**	**Group**		**Pre**	**Post**	**30**	**60**	**90**	**120**	
**CK (U/L)**	C	x	26	319	-	-	-	348	G p = 0.48
		±	101	112				107	T p = 0.00
	S	x	187	244	-	-	-	252	G × T p = 0.67
		±	38	50				61	
	H	x	138	195	-	-	-	184	
		±	21	26				25	
	M	x	196	299	-	-	-	272	
		±	51	69				61	
									
**LDH (U/L)**	C	x	130	187	124	126	133	130	G p = 0.55
		±	8	49	7	5	11	9	T p = 0.10
	S	x	127	189	157	121	124	141	G × T p = 0.95
		±	6	37	30	6	8	21	
	H	x	122	142	131	123	121	120	
		±	5	6	6	8	5	9	
	M	x	132	154	148	125	128	131	
		±	6	9	10	5	6	6	
									
**AST (U/L)**	C	x	22	28	21	22	22	23	G p = 0.35
		±	3	3	2	2	3	2	T p = 0.03
	S	x	22	28	24	21	22	22	G × T p = 0.97
		±	2	3	2	2	2	3	
	H	x	19	22	20	19	19	19	
		±	1	1	1	1	1	1	
	M	x	23	26	24	21	22	22	
		±	2	3	3	2	2	2	
									
**ALT (U/L)**	C	x	14	16	14	14	14	14	G p = 0.25
		±	2	2	2	2	2	2	T p = 0.00
	S	x	16	17	15	15	15	15	G × T p = 0.86
		±	2	2	2	2	2	2	
	H	x	14	16	14	13	13	14	
		±	2	2	1	1	1	2	
	M	x	20	21	20	18	17	19	
		±	2	3	3	2	2	2	

x Represents mean value

± Represents standard error of mean

### General immune markers

Table 5 presents general markers of immunity evaluated in this study. Significant time effects were observed in WBC, neutrophils, and the ratio of total neutrophils to total lymphocytes (p < 0.001). However, no significant group × time interactions were observed.

**Table 5:** General Immune Markers

**Variable**	**Group**		**Pre**	**Post**	**30**	**60**	**90**	**120**	
**WBC **10^9^/L	C	x	5.36	6.44	5.85	6.57	7.23	7.67	G p = 0.79
		±	.41	.84	.64	.62	.70	.68	T p = 0.00
	S	x	5.46	5.91	5.56	7.13	7.63	7.96	G × T p = 0.82
		±	.56	.53	.56	.85	.98	.93	
	H	x	5.95	6.20	6.07	7.80	8.33	9.08	
		±	.49	.61	.54	.93	1.03	1.28	
	M	x	5.29	6.20	5.15	6.40	7.48	7.79	
		±	.24	.56	.35	.59	.80	.90	
									
**Neutrophils **10^9^/L	C	x	3.16	4.57	4.38	5.03	5.68	5.91	G p = 0.57
		±	0.33	0.85	0.59	0.58	0.68	0.67	T p = 0.00
	S	x	2.95	3.22	3.91	5.28	5.57	6.64	G × T p = 0.49
		±	0.41	0.39	0.50	0.82	0.81	0.81	
	H	x	2.93	3.97	4.31	5.97	6.47	7.00	
		±	0.37	0.44	0.56	0.92	0.97	1.19	
	M	x	2.85	3.41	3.26	4.35	5.26	5.07	
		±	0.26	0.46	0.30	0.59	0.80	0.41	
									
**Total Neutrophil/Lymphocyte Ratio**	C	x	1.99	3.79	4.07	4.63	5.86	5.08	G p = 0.24
		±	0.23	0.98	0.58	0.47	1.06	0.67	T p = 0.00
	S	x	1.48	1.55	4.58	4.25	3.70	5.12	G × T p = 0.45
		±	0.15	0.19	1.40	0.89	0.72	1.35	
	H	x	1.68	2.15	3.56	5.63	6.02	5.95	
		±	0.22	0.21	0.75	1.57	1.54	1.47	
	M	x	1.64	1.86	2.12	3.38	3.64	3.64	
		±	0.20	0.36	0.28	0.51	0.49	0.47	

x Represents mean value

± Represents standard error of mean

* p < 0.05 versus control

### Side effects

No significant differences were observed among groups in perceptions of hypoglycemia (p = 0.851), dizziness (p = 0.711), headache (p = 0.422), stomach upset (p = 0.325), or fatigue (p = 0.837).

## Discussion

Ingestion of CHO and PRO following intense exercise has been reported to increase insulin levels, optimize glycogen resynthesis, enhance PRO synthesis, and lessen the immuno-suppressive effects of intense exercise [2,3,8,14,16,35]. Since different forms of CHO have varying glycemic effects [28,29,34], the purpose of this study was to determine whether the type of CHO ingested with PRO following resistance-exercise affects blood glucose availability, insulin levels, markers of anabolism and catabolism, and/or general immune markers during the first two hours of recovery. The major findings of this study were: 1.) ingesting CHO with PRO following resistance-training promoted significant increases in insulin levels; 2.) no significant differences were observed among the forms of CHO ingested on insulin levels suggesting that each of these types of CHO can be an effective source of CHO for post-exercise CHO/PRO supplements; 3.) that glucose levels were maintained to a greater degree in subjects ingesting honey as the source of CHO; and, 4.) post-exercise nutritional supplementation did not significantly affect the time course of testosterone, cortisol, the ratio of testosterone to cortisol, muscle and liver enzyme efflux, or general markers of immunity during the first two hours of recovery following resistance-exercise. These findings add to a growing body of literature indicating that ingestion of CHO and PRO following exercise can stimulate insulin levels and thereby anabolic processes [3,5,12,18,20,27]. Moreover, they extend our understanding of how different sources of CHO with differing glycemic responses influence glucose availability, insulin levels, and recovery indices. The following provides additional insight into the results observed.

Results from the present study indicate that glucose and insulin concentrations peaked 30-min following ingestion of CHO/PRO and then proceeded to decline for 120-min. This finding is of interest from a nutrient delivery and timing standpoint in that it has been suggested that athletes should ingest CHO and PRO within two hours following intense exercise in order to optimize the hormonal effects of intense exercise and recovery [1,36]. It has typically been thought that glucose and insulin levels increase the greatest following ingestion of a high GI form of CHO and that combining high GI carbohydrates with PRO would optimize the insulin and glucose response following exercise. For this reason, post-exercise CHO/PRO supplements often contain dextrose, sucrose, or maltodextrin as the source of CHO. However, it is well known that the GI profile of CHOs may be altered when co-ingesting CHO with PRO, fat, and/or other nutrients due to influences on the energy density, osmolality, and/or gastric emptying rates GI of the meal [30,32,37-40]. Consequently, one can not assume that adding a high GI CHO to a PRO supplement will yield the most advantageous glucose and insulin response. In support of this contention, glucose levels were increased to the greatest degree when ingesting honey as the source of CHO rather than sucrose or maltodextrin. As noted previously, the honey powder used in this study contained fructose (31.5%), glucose (26%), wheat starch (25.3%), and maltose (4.7%). These findings suggest that it may be more advantageous to ingest a mixture of CHO's with PRO following exercise in order to promote a more sustained increase in blood glucose response. However, although ingesting CHO with PRO significantly increased insulin levels in comparison to controls, no significant differences were observe among types of CHO ingested in peak insulin levels (C 8.7 ± 2.9 *u*IU/mL; S 136.2 ± 15.6 *u*IU/mL; H 150.1 ± 25.3 *u*IU/mL; M 154.8 ± 18.9 *u*IU/mL) or AUC values in insulin observed after ingestion of the supplements (C -243 ± 162 *u*IU/mL; S 7,527 ± 883 *u*IU/mL; H 8,656 ± 1,758 *u*IU/mL; M 10,016 ± 974 *u*IU/mL).

It is also interesting to note that glucose values in the H group stayed above baseline throughout recovery while values fell below baseline in the S and M groups. Consequently, this form of CHO may help maintain glucose levels and prevent incidents of hypoglycemia that some individuals may experience when ingesting large amounts of CHO and PRO. Although we did not measure glycogen uptake at the muscle, previous research has shown that ingesting CHO with higher GI following exercise promotes a more rapid resynthesis of muscle glycogen [28,30,41,42]. Additionally, that ingesting CHO and PRO following exercise increases muscle glycogen replenishment [6,8,10]. Since co-ingesting CHO with other nutrients influences the energy density, osmolality, gastric emptying rates, and the GI of the meal [32,37-39], additional research should evaluate the effects of ingesting different forms of CHO with PRO on muscle glycogen resynthesis following intense exercise.

Post-exercise ingestion of PRO and amino acids have been reported to stimulate PRO synthesis [3,12,16,19,26]. Additionally, insulin has been reported to be a potent stimulator of PRO synthesis [3,12,16,18,20]. While there is some debate whether provision of CHO with PRO and/or amino acids enhances the effects on PRO synthesis [43] as well as whether adding PRO or amino acids to CHO promotes greater glycogen resynthesis [44,45], it is clear that individuals engaged in intense training need to ingest these nutrients in order to optimize recovery [1,2,46]. In the present study, we examined the influence of ingesting different forms of CHO with PRO on a number of markers of anabolism and catabolism during recovery in an attempt to determine whether these nutritional strategies influenced the acute phase of recovery. Previous research has indicated that resistance-trained men consuming a CHO:PRO supplement for one week were found to have lower cortisol concentrations during supplementation, as well as higher levels insulin-like growth factor-I following several days of heavy-resistance exercise [15]. Chandler et al. [14] also demonstrated that insulin and growth hormone concentrations during recovery from a single heavy-resistance training session were significantly higher and testosterone concentrations were lower when subjects consumed a CHO:PRO supplement immediately before and 2 h after the workout. The supplements had no effect on IGF-1 but testosterone concentrations decreased and were interpreted by the authors to be the result of increased testosterone clearance [14]. In our trial, testosterone concentrations and the ratio of testosterone to cortisol significantly increased in response to resistance-exercise and then declined during the recovery period. However, the nutritional intervention did not significantly influence this response. Interestingly, cortisol levels increased to the greatest degree in response to exercise in the non-supplemented control group while decreasing in the supplemented groups. However, these apparent differences were not statistically significant. Likewise, significant time effects were observed in creatinine, BUN, the BUN/creatinine ratio, CK, ALT, and AST. However, no significant differences were observed among groups with the exception that the BUN/Creatinine ratio increased significantly during recovery following nutritional supplementation in comparison to the non-supplemented control. The BUN/Creatinine ratio is a general marker of whole body catabolism. Higher levels typically are indicative of greater PRO degradation. However, the increased BUN/Creatinine levels observed following supplementation in the present study were most likely due to the ingestion and utilization of supplemental PRO.

Finally, research has shown that intense exercise causes an acute immuno-suppression for several hours after exercise. For this reason, a number of nutritional countermeasures including CHO and PRO have been proposed to lessen the immunosuppressive effects of intense exercise [1,36]. In this study, we examined whether different forms of CHO influenced general markers of immunity. We found that WBC, neutrophils, and the total neutrophil to total lymphocyte ratio were significantly increased in response to exercise and throughout recovery. These findings support prior findings that intense resistance-training can promote an immune challenge. However, ingestion of CHO and PRO had no influence on these responses during the acute phase of recovery. Whether, ingestion of CHO and PRO may influence markers of immunity during a more prolonged period of recovery remains to be determined.

In conclusion, CHO and PRO ingestion following exercise significantly influences glucose and insulin responses without significantly altering markers of anabolism, catabolism or immunity during the first two hours of recovery. Although ingesting honey as the source of CHO with PRO tended to maintain blood glucose levels to a greater degree, no significant differences were observed among the types of CHO ingested in terms of insulin response to supplementation. These findings suggest that each of these types of CHO can serve as effective sources of CHO to ingest with PRO following intense resistance-exercise in an attempt to optimize CHO availability as well as post-exercise anabolism.

## Competing interests

This study was funded by the National Honey Board (Longmont, CO) under the auspices of the United States Department of Agriculture (USDA).

## Authors' contributions

RBK: Obtained grant, served as PI of study, data analysis, manuscript preparation

CE: Data analysis and manuscript preparation

JL: Study coordinator and data collection

CR: Lab director, data collection

MG: Data collection and analysis

PC: Medical supervision and data collection

ALA: Research design, assisted in obtaining grant funding, data analysis
